# Encapsulation of *Leptadenia pyrotechnica* (Khip) Extract in Carbomer Based Emulgel for Its Enhanced Antioxidant Effects and Its In Vitro Evaluation

**DOI:** 10.3390/gels9120977

**Published:** 2023-12-13

**Authors:** Shamaila Masood, Muhammad Sohail Arshad, Haji Muhammad Shoaib Khan, M. Yasmin Begum, Kashif ur Rehman Khan

**Affiliations:** 1 Department of Pharmaceutics, Faculty of Pharmacy, Bahauddin Zakriya University, Multan 60800, Pakistan; shamailamasood7@gmail.com (S.M.); sohailarshad@bzu.edu.pk (M.S.A.); 2 Department of Pharmaceutics, Faculty of Pharmacy, The Islamia University of Bahawalpur, Bahawalpur 63100, Pakistan; 3 Department of Pharmaceutics, College of Pharmacy, King Khalid University, Abha 61421, Saudi Arabia; 4 Department of Pharmaceutical Chemistry, Faculty of Pharmacy, The Islamia University of Bahawalpur, Bahawalpur 63100, Pakistan; kashifur.rahman@iub.edu.pk

**Keywords:** antioxidant, cosmeceutical, emulgel, HPLC, *Leptadenia pyrotechnica*, phytochemicals

## Abstract

Background: The use of natural products in skin care has been valued for their tremendous therapeutic benefits since ancient times. The current study was aimed at exploring the *Leptadenia pyrotechnica* plant extract and development of a stable emulgel loaded with the same extract to assess its cosmeceutical potentials. Methodology: A stable emulgel loaded with methanolic plant extract along with its control gel was prepared by homogenization. The antioxidant potential of extracts prepared in different solvents (methanol MLP, ethanol ELP, n-hexane nLP, ethyl acetate EALP, and petroleum ether PLP) was determined by DPPH scavenging activity. The presence of phytochemicals was confirmed by total phenolic and flavonoid content analysis (TPC/TFC). HPLC was used for quantification of bioactive components. FTIR analysis was performed for confirmation of functional groups. SPF was calculated via spectroscopic analysis for extract, control gel, and extract loaded emulgel. Stability studies included physical evaluation, pH, conductivity, spreadability, and rheological testing of both control and test emulgels at different temperatures, i.e., 8 °C ± 1, 25 °C ± 1, 40 °C ± 1, 40 °C ± 1 with RH of 75% for a period of 90 days. Results: DPPH radical scavenging activity showed the highest antioxidant activity of 85.5% ± 2.78 for MLP. TPC and TFC were also found to be highest for the methanolic fraction, i.e., 190.98 ± 0.40 mgGAE/g and 128.28 ± 2.64 mgQE/g, respectively. The SPF of methanolic extract, placebo gel, and LPEG was 13.43 ± 0.46, 2.37 ± 0.33, and 7.28 ± 0.56, respectively. HPLC assay confirmed the presence of catechin, vanillic acid, caffeic acid, and sinapinic acid. Rheological analysis showed that formulation has pseudo-plastic flow behavior. Other stability tests also revealed that prepared emulgel is a stable one. Conclusion: A stable emulgel loaded with *Leptadenia pyrotechnica* plant extract was successfully prepared and characterized for its cosmetic effects.

## 1. Introduction

Human skin, as considered to be the largest organ of the body, is very abundantly exposed to stress potentials of the environment [1]. It acts as the protective shield to the underlying tissues against a number of stimuli [2]. These stress factors include microbial growth, solar radiations, temperature fluctuations, and genetic trauma. They all can accumulate to cause skin-related problems, e.g., dryness, aging, infections, sun burn and melanogenesis, etc. Therefore, it needs certain mechanisms to provide skin repair, replenishment, and exfoliation effects [3].

A major cause of aging and other skin-related problems is the production of reactive oxygen species such as free radicals. These free radicals stimulate different mechanisms that ultimately damage the significant macromolecular structures such as proteins, DNA, etc. Thus, the defense system of the body comes under risk, which results in deferred repair of skin injuries [4]. Antioxidants are among the major constituents that help protect the body tissues from the environmental stress and oxidative stress. These may be natural or synthetic ones, endogenous or exogenous, enzymatic or non-enzymatic [5]. Polyphenols and flavonoids are exogenous antioxidants and they are present in different plants, fruits, and vegetables [6]. Application of polyphenols in dermatologic therapy and cosmeceuticals is basically dependent upon their free radical scavenging action. Therefore, plants are used as a source of these bioactive molecules, i.e., phenols and flavonoids [7,8].

*Leptadenia Pyrtechnica* (Family: *Asclepiadaceae*) is a desert shrub that is found mostly in the Mediterranean region, Africa, and Asian countries like west India and the desert region of Pakistan. It is a small shrub that is arrect, leafless, and has numerous bifurcations of aerial parts. It is commonly known as “Khip” in Asian regions. Since primeval times, this plant has been utilized in a number of ailments that include inflammatory diseases, skin diseases, spasmodic disorders, etc. Other effects include antimicrobial, expectorant, diuretic, laxative, and astringent effects [9,10]. Plant sap is very useful for eczema and other skin-related problems, e.g., wounds and infections. It has tremendous free radical scavenging potential. It is rich in phenolic and flavonoid contents, e.g., gallic acid, caffiec acid, epicatechin, quercetin-3-β-d-glucoside, and vannilic acid. Other biologically active chemical constituents include steroidal glycosides, derivatives of polyoxypregnane glycosides, alkloidal contents, kaempferol, and terpenes such as squalene. Different biological activities are associated with these chemicals which involve antimicrobial, anti lipoxygenase, anti tumor, and antifungal activity [9,11].

For cutaneous effects of active plant constituents, there is a need for a delivery vehicle. In addition, we need to consider its physical and sensory characteristics. Emulgels are considered to be the effective carriers for extracts incorporation [12]. Emulgels are emulsion-based gels containing a gellifying agent (e.g., Caropol 934). Their gelling network can entrap drug molecules and thus constitutes a sustained release delivery system. Emulsions are added to the gel network to enhance its cutaneous effects in terms of spreading, absorption, and penetration. Emulsion stability is also improved by combining it with gel [13]. Combining the emulsions with gels has a tremendous quality of loading hydrophilic or lipophilic substances. Therefore, emulgels are among the most suitable systems for cutaneous drug delivery [14].

The aim of the current study was to estimate antioxidant potential of the *Leptadenia pyrtechnica* plant (aerial parts) and formulate an emulgel-containing extract of the aforesaid plant. Further skin rejuvenation effects of this novel drug delivery system, i.e., emulgel, were also discovered by different chemical tests.

## 2. Results and Discussion

### 2.1. Antioxidant Potential (DPPH Scavenging Activity)

**Figure 1:**
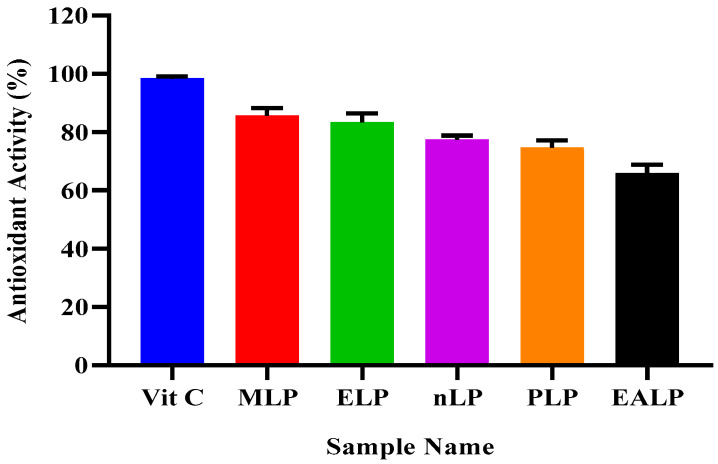
Antioxidant activity of different fractions of LP extract.

By using DPPH Scavenging Assay, antioxidant activities of different LP fractions were determined. It is evident from the results that among all the fractions, methanolic fraction (MLP) showed the highest antioxidant activity, i.e., 85.5% ± 2.78. Other fractions, i.e., ELP, nLP, PLP and EALP showed 83.3% ± 3.06, 77.3% ± 1.52, 73.7% ± 0.89, and 65.7% ± 3.16 of antioxidant activities, respectively, as represented in Figure 1. Ascorbic acid with 98.3% ± 0.77 antioxidant activity was used as a reference standard.

### 2.2. Total Phenolic Contents (TPC)

**Figure 2:**
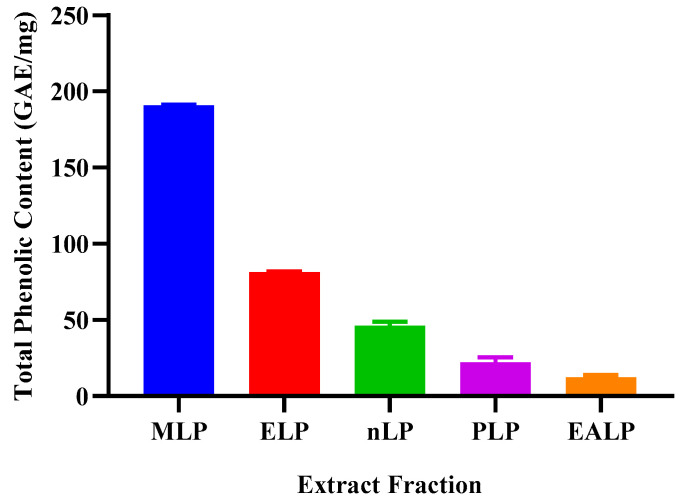
Total phenolic content of different extract fractions.

Total phenolic contents of MLP were found to be the highest as compared to other fractions, i.e., ELP, nLP, PLP and EALP using Folin–Ciocalteu Colorimetric Assay. TPC values of MLP, ELP, nLP, PLP, and EALP were 190.98 ± 0.40 mg, 81.32 ± 0.72 mg, 46.35 ± 2.42 mg, 22.25 ± 3.37 mg, and 12.47 ± 1.44 mg, respectively. Results were indicated in terms of reference standard Gallic Acid (mgGAE/g). As is evident from the results and the graphical illustration (Figure 2), MLP has extracted the highest number of phenols compared to other fractions. Statistical correlation was found to be positive with antioxidant activity (R^2^ = 0.8303).

### 2.3. Total Flavonoid Contents

**Figure 3:**
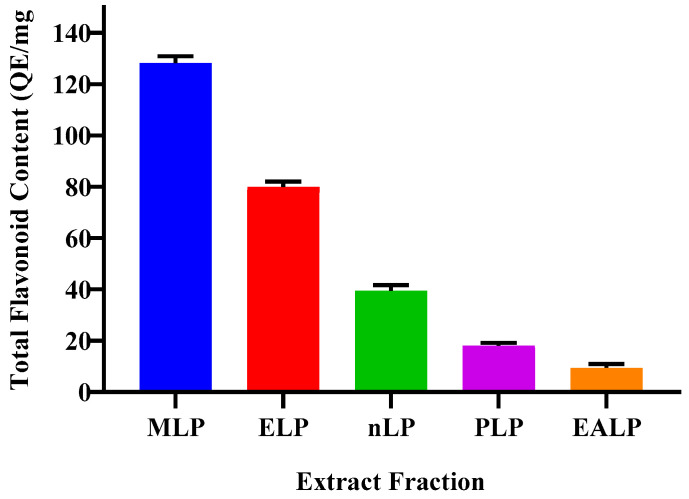
Total flavonoid content of different extract fractions.

Aluminium Chloride Colorimetric Assay was performed for the determination of flavonoids in the LP extract. MLP had 128.28 ± 2.64 mg of flavonoid content, which is the highest among all the fractions. ELP, nLP, PLP and EALP fractions had 79.97 ± 2.05 mg, 39.44 ± 2.29 mg, 18.02 ± 1.12 mg, and 9.48 ± 1.40 mg QE/g, respectively, as depicted in Figure 3. Quercetin solution (1 mg/mL) was used as a standard. The correlation coefficient value was R^2^ = 0.9077, when compared with antioxidant capacity.

### 2.4. Solar Protection Factor

**Figure 4:**
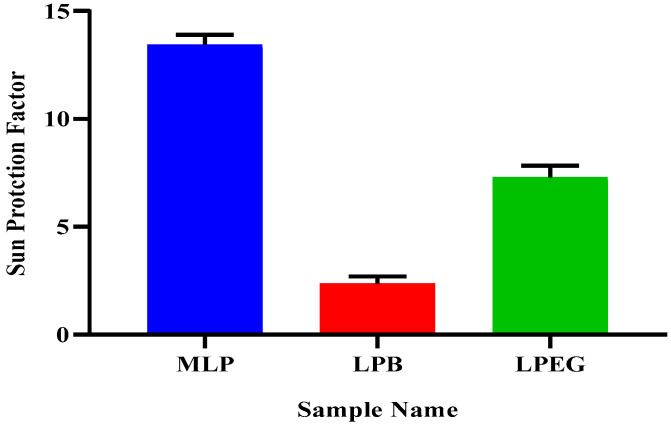
Sun rotection Factor of methanolic extract (MLP), base (LPB), and formulation (LPEG).

Sun protection factor is the quantitative method to determine the capability of a formulation to protect from hazardous effects of UV-rays and subsequent sun burn. In the present study, the SPF of the MLP, the respective formulation, and placebo emulgel was calculated. They showed preeminent sun screen effects with sun protection factor values of 13.43 ± 0.46 for MLP crude extract, 7.28 ± 0.56 for LPEG, and 2.37 ± 0.33 for its respective base as calculated by Mansur’s equation. The placebo gel exhibited negligible protection as compared to crude extract that had a good SPF value. LPEG formulation showed medium protection as shown in Figure 4.

### 2.5. Sensory Evaluation

The physical stability of both the LPEG and placebo gel kept at different temperate conditions (8 °C ± 1, 25 °C ± 1, 40 °C ± 1, 40 °C ± 1 with 75 ± 1% RH) was observed in terms of color, phase separation, and liquefaction. No color change, liquefaction, or phase separation was observed in gels placed at 8 °C and 25 °C over the period of 90 days except for the placebo gel. Its color was slightly changed from white to off-white at the 90th day of observation. A slight change of color for the placebo gel placed at 40 °C and 40 °C + 75% RH had been observed since the 45th day. The color of the LPEG changed from light green to dark green, placed at 40 °C and in 40 °C + 75% RH incubators since day 90 and day 60 of the study, respectively. Slight liquefaction was observed on day 90 in the placebo and LPEG at 40 °C and 40 °C + 75% RH. A slight phase separation had occurred in the placebo gel since the 60th day and in the LPEG on 90th day, kept at 40 °C. A slight phase separation had also been observed since day 60 in both the LPEG and placebo kept at 40 °C + 75% RH. Table 1 shows the physical parameters of the placebo and formulation (LPEG).

### 2.6. pH Determination

**Figure 5:**
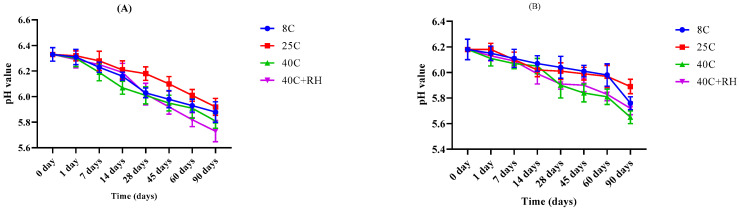
pH variation of the (**A**) base and (**B**) LPEG for 90 days.

pH values of both the placebo and LPEG formulation were noted using a digital pH meter over a period of 90 days for different temperatures, i.e., 8 °C ± 1, 25 °C ± 1, 40 °C ± 1, 40 °C ± 1 with 75 ± 1% RH. A minimum change in the pH of the placebo from 6.33 to 5.88, 5.92, 5.73, and 5.81 was observed at 8 °C ± 1, 25 °C ± 1, 40 °C ± 1, and 40 °C ± 1 with 75 ± 1% RH, respectively. Similarly, there was a slight change in the pH of the LPEG from 6.18 to 5.76, 5.89, 5.65, and 5.72 at 8 °C ± 1, 25 °C ± 1, 40 °C ± 1, and 40 °C ± 1 with 75 ± 1% RH, respectively, as given in Figure 5A,B.

### 2.7. Conductivity

**Figure 6:**
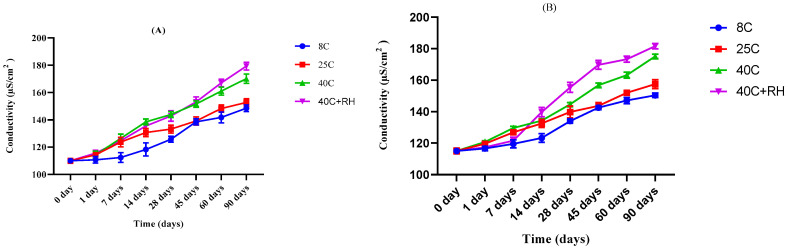
Conductivity of the (**A**) base and (**B**) LPEG for 90 days.

A digital conductivity meter was used for the determination of the conductivity value of the LPEG and placebo that were placed at 8 °C ± 1, 25 °C ± 1, 40 °C ± 1, 40 °C ± 1 with 75 ± 1% RH. Conductivity increased from 110 μS/cm^2^ to 148.5 μS/cm^2^, 152.8 μS/cm^2^, 170.2 μS/cm^2^, and 179.4 μS/cm^2^ for 8 °C, 25 °C, 40 °C, 40 °C + 75% RH, respectively, for placebo emulgel. The values increased from 115 μS/cm^2^ to 150.4 μS/cm^2^, 157.5 μS/cm^2^, 175.1 μS/cm^2^, and 181.6 μS/cm^2^ for LPEG formulation kept at 8 °C, 25 °C, 40 °C, 40 °C + 75% RH, respectively. This increase in conductivity of the placebo gel and LPEG formulation is displayed in Figure 6A,B.

### 2.8. Spreadability

**Figure 7:**
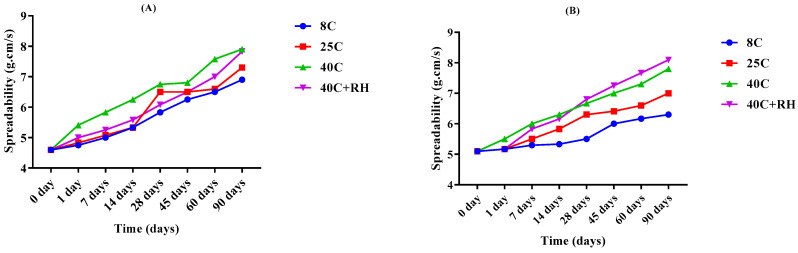
Variation of spreadability of the (**A**) base and (**B**) LPEG for 90 days.

Spreadability was calculated by the method described in the methodology for the placebo and LPEG kept at 8 °C ± 1, 25 °C ± 1, 40 °C ± 1, 40 °C ± 1 with 75 ± 1% RH for 3 months. The values of spreadability increased from 4.6 g.cm/s to 6.9 g.cm/s, 7.3 g.cm/s, 7.9 g.cm/s, and 7.83 g.cm/s on the 90th day for temperature conditions 8 °C, 25 °C, 40 °C, 40 °C + 75% RH, respectively, for the placebo and from 5.1 g.cm/s to 6.3 g.cm/s, 7 g.cm/s, 7.8 g.cm/s, and 8.1 g.cm/s for the LPEG, as illustrated in Figure 7A,B.

### 2.9. Determination of Viscosity

**Figure 8:**
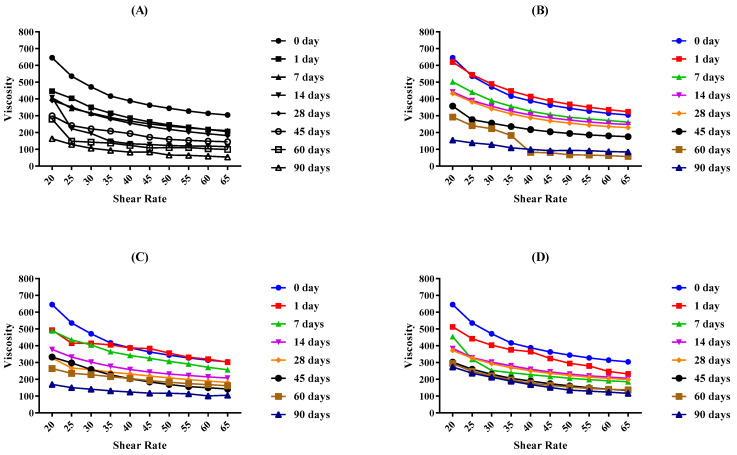
The viscosity of base at (**A**) 8 °C, (**B**) 25 °C, (**C**) 40 °C, and (**D**) 40 °C + RH for 90 days.

**Figure 9:**
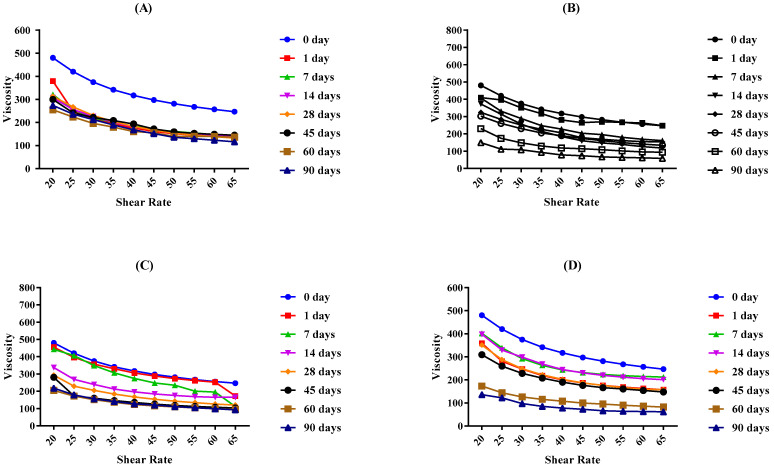
The viscosity of the LPEG at (**A**) 8 °C, (**B**) 25 °C, (**C**) 40 °C, and (**D**) 40 °C + RH for 90 days.

The viscosity of the placebo and LPEG was determined by a programmable Brookfield Rheometer and analytical software RheoCalc 32. Different graphs show that the viscosity of both the LPEG and placebo emulgel increased gradually as the shear stress increased. Changes in the viscosity of the base and LPEG for 90 days stored at different temperatures are depicted in Figure 8 and Figure 9 respectively.

### 2.10. FTIR Analysis

**Figure 10:**
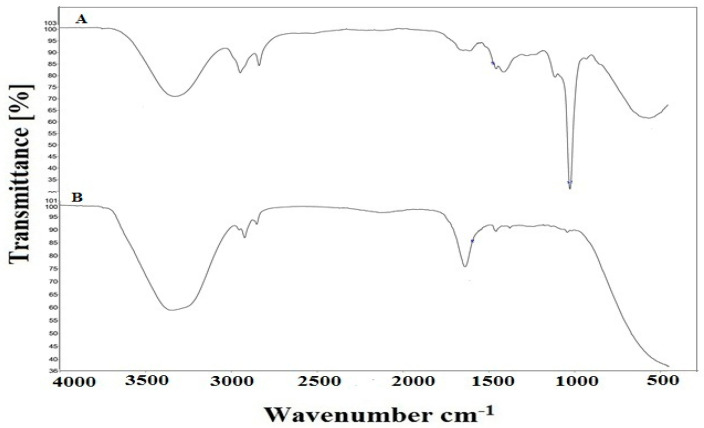
FTIR spectrum of the (A) LP extract and (B) LPEG.

Figure 10 represents the FTIR spectrum of the methanolic extract of the plant and extract containing emulgel, and different frequencies and respective functional groups identified have been reported in the Table 2.

### 2.11. HPLC Analysis

**Figure 11:**
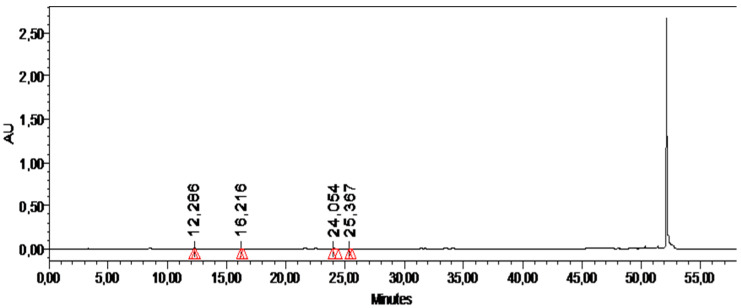
HPLC chromatogram of methanolic plant extract (MLP).

When high performance liquid chromatographic analysis (HPLC) of methanolic plant extract was performed using different analytical standards, different components were identified, namely catechin, vanillic acid, rutin, and sinapinic acid. Their concentrations were 6.07, 1.18, 2.06, and 0.13 µg/mg, respectively. Retention times and concentrations of all detected components are given in Table 3 and illustrated in a chromatogram of the MLP extract (Figure 11).

### 2.12. Discussion

Oxidative stress is increased when the oxidants reach a higher value than the neutralizing species [17]. Antioxidants are the compounds capable of capturing the oxidants, clearing the free radicals from the system, and thus decreasing the oxidative stress. This oxidative stress is responsible for a number of diseases; therefore, it is necessary to overcome the abundance of these free radicals [18,19]. The free radical scavenging property of an extract can be determined by observing the change in color of DPPH from purple to light yellow. The greater the activity, the more the color change [20]. Antioxidants and other phyto-constituents are part of natural therapeutic strategy. This property is affected by macerating the plant material with different solvents. Methanol and ethanol are the most suitable and the best solvents with maximum extraction rate and inclination towards higher polarity [21]. In the present study, plant material was extracted with methanol, ethanol, n-hexane, petroleum ether, and ethyl acetate. More antioxidant activity was exhibited by methanolic extract solution as it is more polar than others. Methanolic and hydro-ethanolic extract had comparable antioxidant activity [19]. Values of TPC and TFC were also higher for the methanolic extract, which supports the statement that more antioxidant potential indicates the high concentration of polyphenolics and flavonoids [19]. Different functional groups that confirm the presence of pregnane glycosides and flavonoid glycosides are responsible for it antioxidant activity, thus helping to counter the oxidative-stress-related diseases [22]. A paired comparison (*t*-test) showed an insignificant change in the values of antioxidant activity and TPC. The value of correlation coefficient (R^2^ = 0.8303) indicates a positive correlation between antioxidant activity and TPC. When the same comparison was made between antioxidant activity and TFC, the change was again insignificant for the *paired sample t*-test. When considering the correlation in terms of R^2^ value (=0.9077), it was elicited that a positive correlation exists between these two variables. The extract with a greater value for antioxidant activity shows greater amounts of TPC and TFC, thus extracting more biochemical components. Therefore, the same extract, i.e., MLP, was used in further studies. Insignificant differences were also found between the values for TPC and TFC of different types of extracts when compared by the application of the *paired sample t*-test. A robust positive correlation with R^2^ = 0.9777 was indicated between these two variables: one increase as the other increases.

The UV radiations when absorbed through the skin epidermis can disturb the oxidative balance of the system. These radiations start producing oxygen and nitrogen free radicals and start a chain reaction that results in different skin-related problems, e.g., skin photoaging, melanoma, and carcinogenesis [23]. Dermatoheliosis is a collective term used for sun-induced damages to the human skin [24]. Solar protection factor is a technique that describes the level of protection provided to the skin by any formulation or pure plant extract [25]. To determine the protection provided by the plant extract used in the present study an invitro method was used. From the result, we can conclude that our plant is a good photo-protective agent, as it had a good SPF value. From the SPF value, we can claim that it provides a high level of sun protection according to US-FDA grading. The SPF of the base and LPEG formulation was also compared and results advocated that the LPEG formulation was a good protective agent against UVR. However, the base showed a lesser value of SPF, so it is concluded that formulation excipients have a minimal impact on the value of SPF.

Organoleptic evaluation of the placebo gel and LPEG was carried out over a period of 90 days at accelerated stability conditions. No color variation of the formulations was detected for formulations kept at 8 °C and 25 °C. But the formulations kept at 40 °C and 40 °C + 75% RH exhibited a little drift towards a darker color, i.e., white to off-white in the case of the placebo gel and light green to dark green in the case of the LPEG. That may be due to the degradation of any of the constituents of the extract [26] or formulation excipients like liquid paraffin found in the oily phase of emulsion [27]. Centrifugation is a primeval parameter that determines formulation stability for long-term storage under stressed conditions. In the case of the current study, there was not any phase separation or liquefaction of the formulations kept at 8 °C and 25 °C. A negligible change in consistency was seen in the emulgels kept at 40 °C and 40 °C + 75% RH. It shows that higher temperatures and humidity increase the kinetic energy of the globules and in turn increase particle collisions [28]. That may lead to slight liquefaction of the formulation that is not very evident in the stored emulgels. It indicates that our formulations (placebo and LPEG) are stable.

The pH of a formulation determines suitability of the formulation for the topical application. If it corresponds to the pH of the skin, i.e., between 4.8–6.6, it appears to be non-toxic and a non-irritant for dermal application. Our placebo and LPEG formulation exhibited a decline in the values of pH with time. It was more evident for 40 °C and 40 °C + RH. This decrease may be due to the liberation of the aldehyde group and organic acids or oxidation that ultimately drags the pH value towards the slightly acidic range [27,29]. The application of Two-Way ANOVA (*p* < 0.05) indicates a significant pH change, rather in the acceptable range of skin pH. Therefore, it can be suitable for topical application without causing any hypersensitivity [30].

Spreadability is defined as “the extent of the area where the emulgel readily spreads when it is applied to the skin” [31]. It is one of the most considerable parameters for patient compliance. It also correlates with pharmacological and physiological effectiveness of the formulation. As spreadability increases, the therapeutic effect of the formulation increases for a certain level. It also depends upon the rheology of the formulation. The lesser the viscosity, the more the spreading occurs, hence the better patient compliance. At higher temperatures, the spreadability coefficient increased rapidly as compared to 8 °C and 25 °C because the viscosity is decreased as the temperature is increased and spreadability is decreased [29]. Statistics, i.e., ANOVA and the paired sample t-test revealed that the change in spreadability was significant, using 95% confidence interval (CI) (*p* < 0.05).

A conductivity test of an emulgel is performed to validate the chemical stability and type of emulgel. In the current study, the conductivity value is above zero, which confirms the emulgel to be an O/W type. Therefore, the external phase, i.e., water, is a good conductor of electricity. There is a change in value of conductivity with time. This change may be due to the migration of water droplets within the emulsion system, which increases the number of free ions in the system, thus increasing the conductivity. A greater increase was observed in the conductivity of the formulations kept at 40 °C and 40 °C + RH, which may be due to high temperature conditions. Application of Two-Way analysis of variance confirmed that these changes were significant at a 95% confidence interval and α = 5% (*p* < 0.05) for different time intervals and temperature conditions [32].

Rheological studies of an emulgel are key parameters to evaluate the formulation stability, spreadability, and therapeutic efficacy [33]. Emulgels with good viscoelastic properties have a better absorption at the site of action [34]. Viscosity of emulgels is also affected by the excipients of the formulation, e.g., liquid paraffin, propylene glycol, surfactants and cosurfactants, etc. A elling agent also plays a pivotal role in the consistency of a gel. As the concentration of the polymer increases, the viscosity also increases [35]. The rheogram study shows that there is an inverse relationship between viscosity and shear rate. As the force applied increases, viscosity decreases. Analysis performed through the application of the Power law describes that both the placebo and LPEG formulation follow pseudo-plastic flow behavior, as the flow index of all the formulations was less than one [33]. Observations from day 0 to day 90 depict a decrease in the viscosity with time for both LPEG and placebo formulation. Also, the temperature factor had a declining effect upon viscosities. From the literature survey, we can deduce that with time, there is a transfer of liquid droplets towards the continuous phase, and as a result, the osmotic pressure increases, thus decreasing the viscosity [29,36].

An analysis of FTIR spectrum was performed to show the compatibility of extract and formulation ingredients. Seven major peaks were eminent from the spectrum of LP extract and five peaks from emulgel loaded with LP extract as represented in the figure. In the FTIR spectrum of the LP extract, a characteristic peak was noted at 3325.03, whichis indicative of OH phenolic group stretching vibrations [37]. Two peaks were observed at 2943.58 and 2833.70, which are characteristic peaks of a saturated methyl group (-CH_3_) of side chains [38]. A peak at 1605.85 represents -C=C stretching vibrations and C=O carbonyl group vibrations in an aromatic ring, [39] and at 1409.18, shows in plane =CH_2_ (aromatic ring) bending [32]. Another peak at 1019.85 represents C-O and C-OH stretching in polysaccharides [40]. A peak is noted at 563.15, which is associated with out of plane -C-OH bending [38]. Similar peaks were seen in the spectrum of the formulation loaded with LP extract at 3351.42, 2924.06, 2854.38, 1636.35, and 1457.11, which are in association with those of the FTIR spectrum of extract and there are no significant differences in both of the graphs. Hence, it can be concluded that formulation excipients are compatible with the extract [27].

HPLC analysis was carried out to confirm the presence of polyphenols and flavonoids in the methanolic extract of the LP plant as well as the identification of the constituents present. Different standard analytes such as gallic acid, quercetin, chlorogenic acid, vanillic acid, syringic acid, 3-OH benzoic acid, 3-OH-4-MeO benzaldehyde, *p*-coumaric acid, sinapinic acid, *t*-cinnamic acid, *p*-OH benzoic acid, *t*-ferulic acid, catechin, naringin, benzoic acid, *o*-coumaric acid, rutin, epicatechin, naringenin, 2,3-diMeO benzoic acid, carvacrol, and harpagoside were used for identification. The HPLC chromatogram of the methanolic extract of the plant showed the four peaks at different retention times, which confirms the presence of different constituents. The polyphenols detected were catechin, vanillic acid, caffeic acid, and sinapinic acid and their retention times were 12.28, 16.21, 24.05, and 25.36 min, respectively. The presence of these constituents has also been verified in the previous studies as well [22]. These antioxidants have a significant role in improving the presentation of the skin by their scavenging action. They impart an anti-melanogenesis effect, are an astringent, have an anti-inflamatory effect, an anti-acne effect, and prevent carcinogenesis by shielding the stratum corneum from solar radiations [41].

## 3. Conclusions

An emulgel containing *Leptadenia pyrotechnica* plant extract was successfully formulated. Analyses showed that it had remarkable antioxidant activity. Its total phenolic and flavonoid contents were ascertained by TPC and TFC; HPLC analysis also identified the key constituents responsible for its cosmetic benefits. The formulation was further passed through different physical storage conditions for its stability analysis via organoleptic, centrifugation, pH conductivity, spreadability, and rheological testing for a three-month study period. Results indicated that emulgel was stable at all storage conditions. It exhibits a good UV protection as it has a very good SPF value. Encapsulation of plant extract into emulgel formulation for topical application can further be evaluated for its biophysical parameters by performing in vivo tests on human skin.

## 4. Materials and Methods

### 4.1. Materials and Equipment

Methanol, ethanol, DPPH (1,1-Diphenyl-2-picrylhydrazyl), Follin–Ciocalteu reagent, propylene glycol, Span 20, Tween 20, Liquid paraffin, sodium hydroxide, Methyl paraben, propyl paraben, Carbopol 940 and Ascorbic acid were purchased from Sigma Aldrich, Burlington, MA, USA.

A rotary evaporator (Heidolph, GmbH & Co., Schwabach, Germany), homogenizer (Euro-Star, IKA D 230, Schönwalde-Glien, Germany), refrigerator (Dawlance, Karachi, Pakistan), UV–VIS spectrophotometer (Bio-Tek Instruments, Bad Friedrichshall, Germany), centrifugation machine (Hettich EBA 20, Bad Bocklet, Germany), pH meter Model (WTW pH-197i, Weilheim, Germany), Digital conductivity meter (WTW COND-197i, Weilheim, Germany), Brookfield Rheometer (RV-DVIII Ultra Rheometer Brookfield Engineering Labs, Stoughton, MA, USA), microplate reader Synergy HT (BioTek Instrument, Winooski, VT, USA) were used.

### 4.2. Methodology

#### 4.2.1. Plant Collection and Identification

*Leptadenia pyrotechnica* (LP) is a desert shrub. Its aerial part was obtained from CIDS (Cholistan Institute of Dessert Studies), The Islamia University of Bahawalpur. It was identified by the expert botanist of Department of Botany, The Islamia University of Bahawalpur. The assigned voucher number is 1156/botany.

#### 4.2.2. Plant Extraction

Drying of plant was carried out under shade and pulverization was performed to obtain fine powder. Then, 20 g of powder was soaked in 400 mL of 70% methanol (70:30) and macerated for 72 h at room temperature. Then, it was filtered with muslin cloth followed by filtration with Whattman’s filter paper no. 40. This filtrate was concentrated to 1/3 of its original volume under a vacuum using a rotary evaporator (Heidolph, GmbH & Co., Schwabach, Germany) at a temperature of 37 ± 3 °C [22]. The extract obtained was named MLP and further extracted with different solvents, i.e., 70% ethanol, n-hexane, petroleum ether, and ethyl acetate by solvent–solvent partitioning and named as ELP, nLP, PLP, EALP. These extracts were then further concentrated at room temperature and kept for storage in a tinted glass container in a refrigerator (4 °C).

#### 4.2.3. Antioxidant Potential (DPPH Scavenging Activity)

The antioxidant activity of different fractions of LP extract was determined by previously described method with slight modification using 0.2 mM DPPH solution. In a test tube, 1 mL of DPPH solution (whose absorbance was adjusted between 0.9–1.1 using methanol) and 1 mL of sample (1 mg/mL) were taken and shaken well. It was then kept in dark for 30 min to allow the reaction to occur. Absorbance was taken at 517 nm using methanol as a negative control and ascorbic acid (1 mg/mL) as a positive control. Three consecutive readings were taken and the mean was calculated [42]. The following Equation (1) was used for calculating the inhibition of DPPH radical.

(1) $$\%age\;inhibition=\frac{\left(Ac-As\right)}{Ac\;}\times 100$$

where, *Ac* = absorbance of control, *As* = absorbance of sample.

#### 4.2.4. Quantification of Phenols (Total Phenolic Content)

Total phenols in the sample were determined by employing Folin–Ciocalteu Colorimetric Assay with slight modifications [43]. An amount of 100 µL of MLP extract (1 mg/mL conc.) was taken in a 96 well plate. An amount of 10 µL of 10% FCR (Folin–Ciocalteu Reagent) was then added and an incubation period of 10 min was given at 37 ± 2 °C temperature. Then, 90 µL of Na_2_CO_3_ was added and the absorbance was recorded at 765 nm by using an automatic microplate absorbance reader (Labtech Model LT-4500, New York, NY, USA). Gallic acid (GA) was used as a positive control. Stock solution of gallic acid (1 mg/mL) was prepared with distilled water. Serial dilutions (10 µg–100 µg) of gallic acid were prepared from this stock and used to construct the calibration curve. Total phenolic contents were calculated as grams of gallic acid equivalents per gram of plant extract (gGAE/g).

#### 4.2.5. Quantification of Flavonoids (Total Flavonoids Content)

Determination of total flavonoids content was conducted by employing the Aluminium Chloride Colorimetric Assay described by [43] with some modifications. Quercetin standard (1 mg/mL) solution was prepared and serially diluted with methanol (10 µg–100 µg). Absorbance of these dilutions was measured by a microplate reader (Labtech Model LT-4500) to generate the calibration curve. This standard curve was used for determination of TPC of the sample. An amount of 100 µL of MLP extract was introduced in a microtiter plate. An amount of 25 µL of sodium nitrite solution (1%) was added to it and kept for 5 min. After that, 10 µL of Aluminium Chloride solution (10%) was added and kept for 5 min. Then, 35 µL sodium hydroxide solution (4%) was added. An amount of 35 µL of absolute methanol was added in the end. Absorbance was recorded at a wavelength of 510 nm. Total flavonoid contents were calculated as grams of quercetin equivalents per gram of the extract (gQE/g).

#### 4.2.6. Formulation of Emulgel

For preparation of the emulgel formulation LPEG, a method used by khan et al. [27] was selected with some modifications. An amount of 2 (%) gel was prepared by wetting 2 g of Carbopol 940 in distilled water for 24 h in the dark and stirred it to make a homogenous gellified network. Its pH was adjusted with triethanolamine, adding drop by drop until it reached 6–6.5. For preparation of emulsion, the aqueous phase was first prepared. Firstly, methyl and propyl paraben were dissolved in propylene glycol and added to distilled water. Then, Tween 20 was added to it. The oily phase contained liquid paraffin and Span 20 (Table 4). Both phases were heated until 75 ± 5 °C. Methanolic extract (MLP) was then added to the liquid phase. Both phases were then mixed slowly with continuous homogenization. Emulsion was poured into the gel (1:1) and homogenized. The speed of the homogenizer was adjusted to 2000 rpm for 15 min, then at 1000 rpm for 10 min, and finally 500 rpm for 5 min during emulsion and emulgel preparation [36]. An identical procedure was employed for preparation of the placebo emulgel LPB.

#### 4.2.7. Solar Protection Factor

Sun protection factor (SPF) evaluation was carried out by the method described by [44] with some modifications. Briefly, 100 mL ethanolic solution of MLP extract (10,000 µg/mL) was prepared and ultrasonicated for 10 ± 5 min. This solution was filtered with Whattman’s filter paper No. 1, discarding first 10 mL. The absorbance spectra of the extract solution were obtained from wavelengths 290–320 nm, with an increment of 5 nm using an Ultraviolet Spectrophotometer. Three measurements were taken at each point. It was followed by an application of Mansur’s equation [45].

(2) $$\mathrm{SPF}=CF\times \sum_{290}^{320}\mathrm{EE}\;\left(\lambda \right)\times I\left(\lambda \right)\times Abs\;\left(\lambda \right)\;$$

where SPF = solar protection factor, EE is the spectrum erythemogenic effect, I = solar intensity, *Abs* = absorbance of MLP extract solution at different wavelengths, and *CF* is the correction factor, i.e., 10 for the given data.

#### 4.2.8. Fourier Transform InfraRed Spectroscopic Analysis

For the identification of different organo-biochemical constituents, FTIR analysis was performed. It identified the components that were actually responsible for the biological and pharmacological activity of the plant that was under investigation. These constituents were recognized on the basis of the type of bonding and their functional groups. A minute concentration of extract solution was placed in the sample chamber with the application of constant pressure. FTIR spectral analysis of formulation was also performed to confirm the compatibility of extract/drug with the formulation excipients. Observations were taken in the wave number range of 400 cm^−1^–4000 cm^−1^.

#### 4.2.9. High Performance Liquid Chromatography (HPLC) Analysis

HPLC analysis, for confirmation of phenolics and flavonoids present in methanolic plant extract, was performed on a Waters liquid chromatograph equipped with a model 600 solvent pump and a 2996 photodiode array detector (PDA), and Empower *v.2* Software (Waters Spa, Milford, MA, USA) was used for acquisition of data. A C_18_ reversed–phase packing column (Prodigy ODS (3), 4.6 × 150 mm, 5 μm; Phenomenex, Torrance, CA, USA) was used for the separation and the column was thermostated at 30 ± 1 °C using a Jetstream2 Plus column oven. The UV/Vis acquisition wavelength was set in the range of 200–500 nm. The quantitative analysis was achieved at maximum wavelength for each compound. The injection volume was 20 μL. The mobile phase was directly online degassed by using Biotech DEGASi, mod. Compact (LabService, Anzola dell’Emilia, Italy).

An amount of 22 standard compounds were employed for an eluting purpose as analytes, namely epicatechin, gallic acid, chlorogenic acid, catechin, vanillic acid, 3-OH benzoic acid, 2,3-diMeO benzoic acid, syringic acid, 3-OH-4-MeO benzaldehyde, *p*-coumaric acid, *p*-OH benzoic acid, rutin, sinapinic acid, *t*-ferulic acid, *t*-cinnamic acid, naringin, benzoic acid, *o*-coumaric acid, quercetin, harpagoside, naringenin, and carvacrol. All samples were prepared as follows: plant extracts were weighed on an analytical balance and solubilized in mobile phase A (milliQ water + 3% acetic acid): B (acetonitrile +3% acetic acid) (93:7, *v*:*v*). Samples were prepared at a concentration of 1 mg/500 µL. All samples were agitated through vortex mixing for 30 s, sonicated for 15 min, and centrifuged for 10 min. Supernatants were injected (20 µL) in HPLC system for the analysis. Sample run time was 60 min. Gradient elution was 93% of A and 7% of B at 0 time, then it changed to 72% of A over 30 min, 75:25 during (30–38 min), and finally to the initial concentration during 38–48 min. It retained that point for 10 min further. The flow rate was kept 1 mL/minute over th period of time. The peaks identified on the chromatogram were then brought to comparison with the compounds present in the HPLC library for the ascertainment of the polyphenol and flavonoid compounds.

#### 4.2.10. Stability Studies of Placebo LPB and Extract Based Emulgel LPEG

The physical stability of the LPB and LPEG was evaluated during three months. Measurements were taken on 0 days, 1 day, 7th day, 14th day, 28th day, 45th day, 60th day, and 90th day. Temperature conditions set for the following study were 8 °C ± 1, 25 °C ± 1, 40 °C ± 1, 40 °C ± 1 with 75 ± 1% RH (relative humidity). Formulations were evaluated for following study parameters.

##### Sensory Evaluation

Changes in color, physical appearance, phase separation, liquefaction of LPB and LPEG kept at 8 °C ± 1, 25 °C ± 1, 40 °C ± 1, 40 °C ± 1 with 75 ± 1% RH (relative humidity) were evaluated during a 3-month period. Phase separation and liquefaction were observed with a centrifugation test [46]. An amount of 10 mg of the emulgel was taken in a test tube and placed in the centrifugation machine (Hettich EBA 20, Germany). The machine was run at 3500 rpm for 15 min and then emulgel was allowed to stand and checked for any signs of instability.

##### pH Determination

The pH of prepared LP extract-based emulgel (LPEG) and its placebo formulation kept at different storage conditions was taken at different time intervals during a period of 90 days. pH meter Model (WTW pH-197i, Germany) was used. Three consecutive readings were taken and the mean was calculated.

##### Conductivity Measurement

Conductivity of prepared LP extract-based emulgel (LPEG) and its placebo formulation kept at different storage conditions was taken at different time intervals during a period of 90 days. Three consecutive readings were taken and the mean was calculated. Digital conductivity meter (WTW COND-197i, Germany) was used for this determination and readings were taken at room temperature, i.e., 25 °C.

##### Spreadability

Spreadability test of placebo emulgel and LPEG was performed for a 90-day period. For this purpose, protocol used by [47] was applied with some modifications. An amount of 500 mg of emulgel was applied on a glass slide in an area of 1 cm. Another glass slide was allowed to rest in parallel of the slide below and a weight of 500 g was put on it for 5 min. Spreadability was measured by the formula [48] as follows

(3) $$Sp=m\frac{l}{t}$$

where S*p* represents spreadability, *m* = weight on upper slide (g), *l* = length of spreading (cm), and *t* = time allowed for spreading (s).

##### Determination of Viscosity

Different rheological parameters of LPEG and its respective control formulation were determined with the method used by [32] making a few modifications. A programmable Brookfield Rheometer (RV-DVIII Ultra Rheometer Brookfield engineering labs. USA) with software Rheocalc Version 2.6 as a support program was used for data analysis. A spindle number CP-41 with (5–50 rpm) speed and shear rate (10–100) was selected. An amount of 0.2 ± 0.1 g of formulation was applied on the plate of the Rheometer and viscosities were recorded at 25 ± 0.5 °C. Measurements were taken at intervals of days 0, 1, 7, 14, 28, 45, 60, and 90.

#### 4.2.11. Statistical Analysis

Results were statistically and scientifically analyed by different statistical tools. The program named SPSS (Statistical Package for Social Sciences) was used for applying different statistical tools. For interpretation of our study results, two-way ANOVA was used along with Student’s *paired sample t*-test. *Post hoc* analysis via least significant difference (LSD) was applied to perform the paired comparison. The level of significance for all of these analyses was set at 5% (*p* = 0.05).

## Figures and Tables

**Table 1:** Sensory evaluation of the LP-based emulgel and base formulation.

Parameter	Temperature	8 °C		25 °C		40 °C		40 °C + 75% RH	
	Time	B	F_L_	B	F_L_	B	F_L_	B	F_L_
Color	Fresh	W	LG	w	LG	w	LG	W	LG
	Day 1	W	LG	w	LG	w	LG	W	LG
	Day 7	W	LG	w	LG	w	LG	W	LG
	Day 14	W	LG	w	LG	w	LG	W	LG
	Day 28	W	LG	w	LG	w	LG	W	LG
	Day 45	w	LG	w	LG	Ow	LG	Ow	LG
	Day 60	w	LG	w	LG	Ow	LG	Ow	DG
	Day 90	w	LG	Ow	LG	Ow	DG	Ow	DG
Liquefaction	Fresh	-	-	-	-	-	-	-	-
	Day 1	-	-	-	-	-	-	-	-
	Day 7	-	-	-	-	-	-	-	-
	Day 14	-	-	-	-	-	-	-	-
	Day 28	-	-	-	-	-	-	-	-
	Day 45	-	-	-	-	-	-	-	-
	Day 60	-	-	-	-	-	-	-	-
	Day 90	-	-	-	-	S	S	S	S
Phase separation	Fresh	No	No	No	No	No	No	No	No
	Day 1	No	No	No	No	No	No	No	No
	Day 7	No	No	No	No	No	No	No	No
	Day 14	No	No	No	No	No	No	No	No
	Day 28	No	No	No	No	No	No	No	No
	Day 45	No	No	No	No	No	No	No	No
	Day 60	No	No	No	No	S	No	S	S
	Day 90	No	No	No	No	S	S	S	S

B = Base, F_L_ = formulation. - = no, S = slight. w = white, Ow = off-white, LG = light green, DG = dark green.

**Table 2:** Interpretation of the FTIR spectrum of the plant extract.

Peak Value		Functional Groups	Frequency Range	Bonds	Secondary Metabolites
Plant Extract	LPEG Formulation				
	3351.42	Alcoholic and phenolic hydroxyl groups	3200–3600	-O-H	Alkaloids, flavonoids, tannins, saponins, polyphenols, steroids, terpenoids, carboxylic acid containing phytochemicals, etc.
3325.03		Alcoholic and phenolic hydroxyl groups	3200–3600	-O-H	
2943.58		Alkanes	2850–2970	-C-H, -CH_3_	
	2924.06	Alkanes	2850–2970	-C-H, -CH_3_	
	2854.38	Alkanes	2850–2970	-C-H, -CH_3_	
2833.70		Alkanes	2850–2970	-C-H, CH_3_	
	1636.35	Secondary amines, Aldehydes, ketones, carboxylic acid esters	1550–1650	-N-H -C=C	
1605.85		Secondary amines Aldehydes, ketones, carboxylic acid esters	1550–1650	-N-H -C=O	
	1457.11		1340–1470	=CH_2_	
1409.18			1340–1470	=CH_2_	
1019.85			1020–1090	C-O and C-OH	
563.15				C-H bending	

These frequencies and respective functional groups have been cited from previously available data [15,16].

**Table 3:** HPLC analysis of the methanolic extract of the plant, compounds identified, and their retention times with concentrations (µg/g).

Compounds Detected	RT for STD	MLP Conc. (µg/mg)	MLP RT (min)
Catechin	12.11	6.07	12.26
Vanillic acid	15.09	1.18	15.21
Rutin	24.15	2.06	24.04
Sinapinic Acid	25.21	0.13	25.36
Total		9.45	

MLP = methanolic plant extract, RT = retention time.

**Table 4:** Composition of placebo (LPB) and LPEG formulation.

Ingredients (*w*/*w*)	LPB (g)	LPEG (g)
Emulsion		
Liquid paraffin	7.5	7.5
Span 20	0.5	0.5
Tween 20	1	1
Propylene glycol	5	5
Methyl paraben	0.03	0.03
Propyl paraben	0.01	0.01
MLP Extract (1 mg/mL)	-	2
Distilled water q.s	100	100
**Gel**		
Carbopl 940	2	2
Triethanolamine Distilled water q.s	Few drops 100	Few drops 100

## Data Availability

The data used to support the findings of this study are included within the article.
